# Risk of Dementia in Older Patients with Type 2 Diabetes on Dipeptidyl-Peptidase IV Inhibitors Versus Sulfonylureas: A Real-World Population-Based Cohort Study

**DOI:** 10.3390/jcm8010028

**Published:** 2018-12-28

**Authors:** Young-Gun Kim, Ja Young Jeon, Hae Jin Kim, Dae Jung Kim, Kwan-Woo Lee, So Young Moon, Seung Jin Han

**Affiliations:** 1 Department of Medical Sciences, Ajou University Graduate School, Suwon 16499, Korea; ygkim25@gmail.com; 2 Ministry of Health and Welfare, Gyeonggi Provincial Government, Suwon 16444, Korea; 3 Department of Endocrinology and Metabolism, Ajou University School of Medicine, Suwon 16499, Korea; Twinstwins@hanmail.net (J.Y.J.); jinkim@ajou.ac.kr (H.J.K.); djkim@ajou.ac.kr (D.J.K.); LKW65@ajou.ac.kr (K.-W.L.); 4 Department of Neurology, Ajou University School of Medicine, Suwon 16499, Korea; symoon.bv@gmail.com

**Keywords:** dementia, dipeptidyl-peptidase IV inhibitors, diabetes mellitus, type 2, Alzheimer’s disease, dementia, vascular

## Abstract

Background: Type 2 diabetes is related to an increased risk of dementia. Preclinical studies of dipeptidyl peptidase-IV inhibitors (DPP-4i) for dementia have yielded promising results. Therefore, we investigated the risk of dementia in elderly patients with type 2 diabetes on DPP-4is and sulfonylureas (SU). Methods: Using a claims database called the Korean National Health Insurance Service Senior cohort, new users of DPP-4is and SUs were matched by 1:1 propensity score matching using 49 confounding variables (7552 new DPP-4is users and 7552 new SU users were matched by 1:1 propensity score matching; average age 75.4; mean follow-up period: 1361.9 days). Survival analysis was performed to estimate the risk of dementia. Results: The risk of all-cause dementia was lower in the DPP-4i group compared to the SU group (hazard ratio (HR) 0.66; 95% confidence interval (CI) 0.56–0.78; *p* < 0.001). Particularly, DPP-4i use showed a significantly lower risk of Alzheimer’s disease (HR 0.64; 95% CI 0.52–0.79; *p* < 0.001) and a lower risk, albeit non-significant, of vascular dementia compared to SU use (HR 0.66; 95% CI 0.38–1.14; *p* = 0.139). Conclusion: Our findings suggest that DPP-4i use decreases the risk of dementia compared to SU use in elderly patients with type 2 diabetes in a real-world clinical setting.

## 1. Introduction

Type 2 diabetes and dementia are prevalent in the elderly and have considerable impacts on public health and patient quality of life. Recent estimates suggest that 382 million and 44 million individuals worldwide are affected by type 2 diabetes and dementia, respectively [1,2]. Epidemiological evidence indicates that diabetes is associated with an increased risk of dementia, including Alzheimer’s disease and vascular dementia [3,4]. According to a recent meta-analysis of 28 prospective observational studies, patients with diabetes have a 73% higher risk of dementia compared to those without diabetes [5]. Although interventions to prevent and treat the classical macro- and microvascular complications of diabetes have improved, cognitive dysfunction and dementia are emerging as important complications in a rapidly aging society [6].

Type 2 diabetes shares several pathophysiological components with dementia, such as glucotoxicity, insulin resistance, inflammation, and oxidative stress [7]. These similarities suggest that anti-diabetic medications may be effective against dementia. Preclinical and clinical studies have investigated the effects of glucose-lowering agents on dementia and cognitive dysfunction but have reported inconsistent results [8,9].

Dipeptidyl peptidase-4 inhibitors (DPP-4i) are widely used oral hypoglycemic agents associated with a low risk of hypoglycemia and weight gain [10]. DPP-4is improve glucose metabolism by increasing the bioavailability of active glucagon-like peptide-1 by inhibiting its degradation. DPP-4is also have neuroprotective, anti-inflammatory, and anti-atherosclerotic effects. Moreover, DPP-4is attenuated amyloid-β deposition and tau phosphorylation in streptozotocin induced Alzheimer’s disease model [11,12]. A DPP-4i also improved memory and learning impairment, brain inflammation, and endothelial dysfunction in a pancreatectomy-induced diabetes model [13].

In a recent cross-sectional study, higher DPP-4 plasma activity was associated with an increased risk of mild cognitive impairment in elderly patients with type 2 diabetes [14]. This suggests that DPP-4is may be effective against cognitive dysfunction in individuals with type 2 diabetes. However, to our knowledge, no clinical study on the effect of DPP-4is on the incidence of dementia in type 2 diabetes has been reported. As long-term use of sulfonylurea (SU) was not associated with an increased risk of Alzheimer’s disease in a population-based case-control study [15], we investigated the risk of dementia in older patients on DPP-4is compared with SUs in a population-based cohort study using a national health insurance database.

## 2. Methods

### 2.1. Study Design and Data Source

We conducted a population-based retrospective observational cohort study using the Korean National Health Insurance Service Senior cohort (ver. 3.0, 1 January, 2002 to 31 December, 2015), which comprises 550,000 (10%) individuals of the South Korean population >60 years of age as of 2002. The database was created using a stratified random sampling method with 1476 strata and is thus representative of the Korean senior population. It contains information on demographic characteristics, socioeconomic status, and claims, such as diagnosis (International Classification of Diseases, 10th revision (ICD-10) code), drug prescriptions, and medical procedures. Socioeconomic status was indirectly assessed using the annual medical insurance premium determined based on the participant’s income and assets, such as property and automobile ownership. Socioeconomic status was defined by dividing medical insurance premiums into 11 quantiles. This study was approved by the Institutional Review Board of Ajou University Hospital (AJIRB-MED-EXP-18-033), which waived the requirement for informed consent because all patient data were de-identified.

### 2.2. Inclusion and Exclusion Criteria

**Figure 1:**
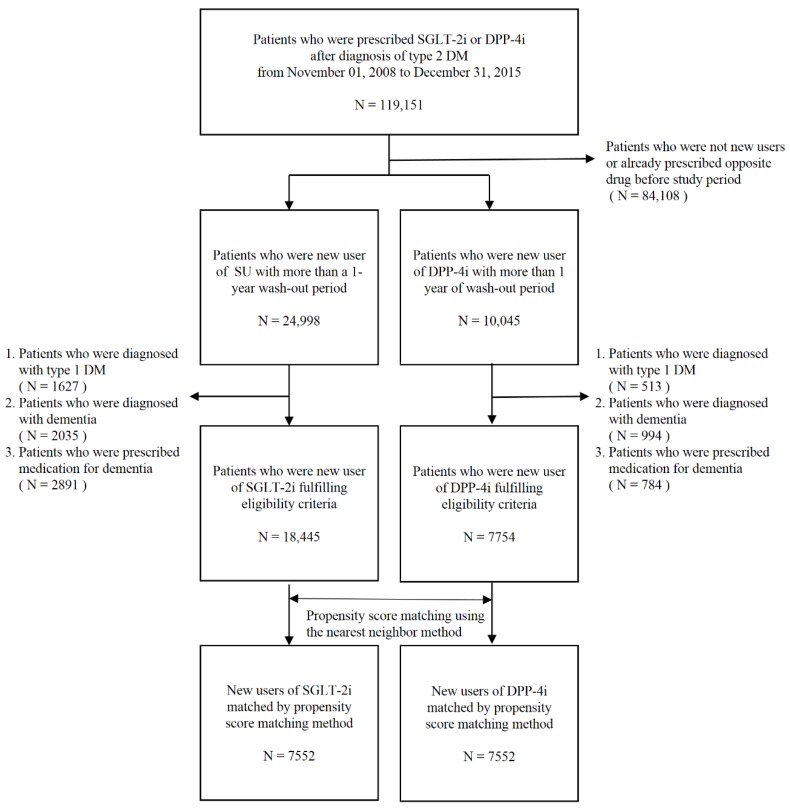
Flow chart of the sample selection process. DM, diabetes mellitus; DPP-4i, dipeptidyl-peptidase IV inhibitor; N, number; SGLT-2i, sodium-glucose co-transporter 2 inhibitor; SU, sulfonylurea.

Patients were included in the cohort if they were aged >60 years with type 2 diabetes and started taking a DPP-4i or SU from 1 November, 2008 to 31 December, 2015, regardless of whether they were taking other hypoglycemic agents (DPP-4is were first approved in Korea on 1 November, 2008). Patients who used both drugs were excluded. A 1-year wash-out period before the first prescription of an SU or DPP-4i enabled identification of new users of each drug type. The prescribed drug and date were defined as the index drug and index date, respectively. Patients who had been diagnosed with type 1 diabetes mellitus or dementia before the index date or who had been prescribed donepezil, memantin, rivastigmine, or galantamine for dementia were excluded. A flowchart of the patient selection process is presented in Figure 1. The follow-up period was calculated from the index date to the first occurrence of study outcomes or the study end date (31 December, 2015).

### 2.3. Study Outcome and Subgroup Analysis

The primary outcome was the first diagnosis of all-cause dementia (ICD-10 codes: F00, F01, F02, F03, F04, F05, G30, or G31), and the secondary outcomes were the first diagnosis of Alzheimer’s disease (F00, G30) or vascular dementia (F01). Subgroup analyses were performed according to sex, age (<75 and ≥75 years), and the presence of DM microvascular or macrovascular complications. DM microvascular complications were defined as at least one of DM nephropathy, neuropathy, or retinopathy, and DM macrovascular complications as at least one of stroke, transient ischemic attack, acute myocardial infarction, other ischemic heart disease, and peripheral artery occlusive disease.

### 2.4. Statistical Analysis

R software (ver. 3.3.3; R Development Core Team, Vienna, Austria) and SAS (ver. 9.4; SAS Institute, Cary, NC, USA) were used for statistical analyses. Data are expressed as means ± standard deviation. The primary method of statistical adjustment was propensity score matching. Among the patients who met the inclusion/exclusion criteria mentioned above, patients with similar characteristics were selected at a ratio of 1:1 from both groups using propensity score matching. We used the nearest-neighbor technique with a caliper of 0.1 on the probability scale, and replacement of the control was not permitted. The following variables (Table 1): age, sex, socioeconomic status (index date), diagnoses (1 year before the index date), and prescribed drugs (180 days before the index date) were used to calculate propensity scores, and thus those variables were adjusted. Because the claims database does not contain information on the duration of diabetes, we adjusted for several variables that could indirectly reflect disease duration, such as diagnostic codes for DM triopathy, acute myocardial infarction, other ischemic heart diseases, ischemic stroke, hemorrhagic stroke, transient ischemic attack, and peripheral artery occlusive disease, as well as prescriptions for other hypoglycemic agents, including insulin. The quality of correction of confounding variables between the two groups was evaluated as a standardized difference. An absolute standardized difference between groups of <0.1 was considered negligible. After propensity score matching, survival analyses were performed among matched pairs to evaluate the effect of DPP-4is on dementia using the one minus survival probability computed by the Kaplan-Meier approach. As several confounding variables were adjusted for by propensity score matching, univariate Cox regression analysis was performed.

## 3. Results

The cohort comprised 18,445 new SU users and 7754 new DPP-4i users, for a total of 12,833 person-years. After propensity score matching, 7552 pairs remained. The mean follow-up period of the matched pairs was 1361.9 days. Approximately 94, 37, and 15% of patients were already prescribed metformin, insulin, and alpha-glucosidase inhibitors, respectively. Table 1 lists the other baseline characteristics of the matched pairs. The standardized differences of all variables were less than 10%, and the mean standardized difference was 1.04% (1.03%). Thus, the baseline characteristics of the matched pairs were well adjusted.

**Figure 2:**
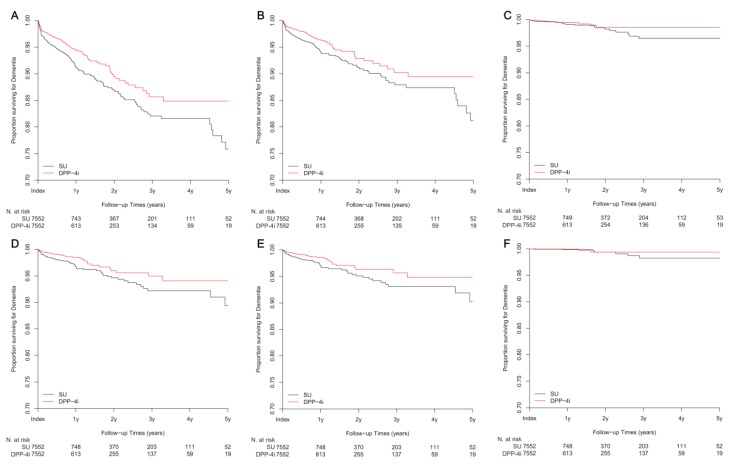
Kaplan-Meier plots for dementia-free survival in new users of DPP-4i and SU. (**A**–**C**) Dementia was defined by diagnosis codes; all-cause dementia (**A**), Alzheimer’s disease (**B**), vascular dementia (**C**). (**D–F**) Dementia was defined by both diagnosis codes and medications; all-cause dementia (**D**), Alzheimer’s disease (**E**), and vascular dementia (**F**). DPP-4i, dipeptidyl peptidase-4 inhibitor; *N*, number of patients; SU, sulfonylurea; y, year(s).

During the study period, 565 patients had newly developed dementia, among whom 367 had Alzheimer’s disease and 54 vascular dementia. When dementia was defined by diagnosis codes, the risk of all-cause dementia was lower in the DPP-4i group compared to the SU group (Figure 2A and Table 2; hazard ratio (HR) 0.66; 95% confidence interval (CI) 0.56–0.78; *p* < 0.001). Additionally, the risk of Alzheimer’s disease was significantly lower in the DPP-4i group (Figure 2B, Table 2; HR 0.64; 95% CI 0.52–0.79; *p* < 0.001). The DPP-4i group also had a lower risk, albeit non-significant, of vascular dementia (Figure 2C, Table 2; HR 0.66; 95% CI 0.38–1.14; *p* = 0.14). Furthermore, when dementia was defined using both diagnosis codes and medications, similar trends were observed; that is, the DPP-4i group also had a lower risk of all-cause dementia and Alzheimer’s disease (Figure 2D–F and Table 2; HR 0.54; 95% CI 0.40–0.73; *p* < 0.001 for all-cause dementia, HR 0.54; 95% CI 0.39–0.75; *p* < 0.001 for Alzheimer’s disease, HR 0.46; 95% CI 0.14–1.46; *p* = 0.18 for vascular dementia).

Subgroup analyses were performed to determine whether age, sex, and DM complications influenced the protective effect of DPP-4i against dementia (Table 3). DPP-4i use was significantly associated with a lower risk of dementia in males and females. DPP-4i use was associated with a lower risk of dementia in patients aged ≥75 years (HR 0.61; 95% CI 0.50–0.76; *p* < 0.001) but not in those aged <75 years (HR 0.77; 95% CI 0.58–1.03; *p* = 0.08), compared to SU use. Patients without diabetic microvascular complications had a significantly lower HR for dementia in the DPP-4i group compared to the SU group (HR 0.64; 95% CI 0.52–0.78; *p* < 0.001). Among patients with diabetic microvascular complications, DPP-4i use was not significantly associated with an improvement in dementia (HR 0.74; 95% CI 0.53–1.03; *p* = 0.07). However, compared with SU use, DPP-4i use was associated with a lower risk of dementia irrespective of diabetic macrovascular complications.

## 4. Discussion

This population-based study demonstrated that use of DPP-4i was associated with a 34% lower risk of all-cause dementia compared with use of SUs in older patients with type 2 diabetes. Indeed, DPP-4i use was related to a significantly lower risk of Alzheimer’s disease, but not vascular dementia, compared with SU use.

To our knowledge, this is the first report that DPP-4i use is associated with a lower risk of dementia in older patients with type 2 diabetes. Our cohort was large and representative of the Korean senior population, enabling propensity score-matched analyses. We also used a new-user design with a one-year washout period to reduce the bias inherent in retrospective nonrandomized comparative effectiveness studies.

Insulin resistance and impaired insulin signaling due to chronic hyperglycemia in the brain may induce hyperphosphorylation of tau protein and accumulation of amyloid-β protein, which are hallmarks of Alzheimer’s disease [8,16]. In addition, cerebrovascular diseases such as stroke, which are prevalent in diabetes, are closely associated with the development of vascular dementia and the progression of Alzheimer’s disease. Because there are interactions between diabetes and dementia and there is no curative treatment for dementia, the effects of antidiabetic medications on cognitive function are of interest.

Our findings support previous reports of a neuroprotective effect of DPP-4is. Research has shown that in human neurons, linagliptin alleviates amyloid-β-induced impaired insulin signaling and neurotoxicity [17]. Long-term sitagliptin treatment attenuated memory impairment and reduced inflammation, nitrosative stress, and amyloid-β protein and amyloid precursor protein accumulation in the brains of transgenic mice with Alzheimer’s disease [18]. Vildagliptin and sitagliptin reversed mitochondrial dysfunction in the brain by decreasing mitochondrial reactive oxygen species production and insulin signaling, and improved the learning and memory deficits induced by high-fat-diet consumption [19,20]. In addition, sitagliptin treatment improved memory impairment in mice fed a high-fat diet by enhancing hippocampal neurogenesis and reducing oxidative stress [21]. In a streptozotocin-induced rat model of Alzheimer’s disease, saxaglitpin and vildagliptin decreased amyloid-β deposition and tau phosphorylation by increasing hippocampal glucagon-like peptide-1 levels, which reversed the cognitive deficits [11,12]. However, DPP-4is reportedly increases the risk of Alzheimer’s disease by aggravating tau phosphorylation and insulin resistance in the hippocampus and primary neurons of OLEF (Otsuka Long-Evans Tokushima Fatty) rats [22].

Few clinical studies have addressed the association between DPP-4is and cognitive function in type 2 diabetic patients. In a prospective pilot study, 10 older patients with type 2 diabetes treated with vildagliptin together with metformin exhibited no cognitive decrements after a 1-year follow up [23]. Furthermore, some previous studies have shown that DPP-4i not only protects against cognitive impairment, but also acts as a cognitive enhancer. Rizzo et al [24]. reported that DPP-4is improved cognitive function compared with SUs, independently of sustained chronic hyperglycemia and glucose variability, in 240 older patients with type 2 diabetes and mild cognitive impairment. In addition, sitagliptin treatment for six months was associated with an increase in the Mini-Mental State Examination score (independent of the change in HbA1c level) compared with metformin treatment in older diabetic patients with or without Alzheimer’s disease [25]. These results suggest that DPP-4is could be a cognitive enhancer or protect against cognitive impairment while also functioning as an anti-diabetic agent, which may explain its effects on the risk of dementia. However, these studies had limitations due to a small sample size and short duration of follow-up. The current results are consistent with previous clinical research that reported the beneficial effects of DPP-4is on cognitive function. As our study included a large older population with type 2 diabetes (mean age 75 years) who had a high risk of dementia in a real-world clinical setting, we believe that these findings provide evidence of the protective effects of DPP-4i on the incidence of dementia.

DPP-4i use was associated with a lower risk of Alzheimer’s disease, but not vascular dementia, compared with SU use. This finding implies that the efficacy of DPP-4is varies among the types of dementia. Meta-analyses of three large cardiovascular outcome trials of DPP-4i (the SAVOR-TIMI 53, EXAMINE, and TECOS trials) as well as a pooled analysis of small randomized clinical trials showed no significant difference in the risk of stroke between DPP-4i and placebo treatments [26]. Considering these neutral effects of DPP-4i on the risk of stroke, which is a predisposing factor for vascular dementia, DPP-4is may not protect against vascular dementia.

In our subgroup analysis, the association between DPP-4i use and a decreased risk of dementia was not evident in patients aged <75 years or in those with diabetic microvascular complications. Although DPP-4i use was related to a lower risk of dementia in subjects with and without diabetic macrovascular complications, the association was weaker in those with diabetic macrovascular complications. Therefore, the protective effect of DPP-4is against dementia may be greater in older patients and those without diabetic complications.

This study had several limitations. This study was a retrospective analysis, and the claims database lacked information on patient medical histories (most notably, DM duration and body mass index (BMI)), education, lifestyle variables, and laboratory measurements (such as HbA1c); therefore, confounding factors may have influenced the results. Randomized clinical trials on how DPP-4is affects the incidence of dementia are needed to confirm our results. The ongoing CAROLINA-cognition sub-study is exploring whether DPP-4is are superior to SUs in terms of preventing cognitive decline in patients with type 2 diabetes [27]. Additionally, we calculated the incidence of dementia according to diagnosis codes; thus, discrepancies between the medical diagnosis and the diagnosis in the claims data may have reduced the accuracy of the analysis [28]. According to a previous study reporting the accuracy of dementia diagnosis code in Medicare claims data in regard to clinically-diagnosed dementia, the sensitivity and specificity of dementia diagnosis codes in the claims database were 0.85 and 0.89, respectively [29]. When we performed additional survival analyses for dementia defined by both diagnosis codes and prescriptions for dementia, the results showed similar trends. In particular, patients with mild cognitive impairment are less detectable in retrospective observational studies performed using claims databases. Finally, only Koreans were analyzed in this study; therefore, caution should be used when generalizing our results to other ethnicities.

In conclusion, compared with SU use, DPP-4i use was associated with a lower risk of dementia in older Koreans with type 2 diabetes. Further research in other populations using dementia as an endpoint is needed to further assess the neuroprotective effects of DPP-4is.

## Figures and Tables

**Table 1:** Baseline characteristics of the matched pairs.

	SU	DPP-4i	SMD
*N*	7552	7552	
Age (SD)	75.42 (5.31)	75.39 (4.73)	0.007
Sex (Male, percent)	44.01	43.39	0.013
Socio-economic status (*n*, (%))			0.060
1st to 4th of 11 quantiles	1892 (25.05)	1892 (25.05)	
5th to 8th of 11 quantiles	2301 (30.47)	2378 (31.49)	
9th to 11th of 11 quantiles	3359 (44.48)	3282 (43.46)	
Hypertension	79.61	80.39	0.020
Dyslipidemia	74.13	74.44	0.007
Chronic kidney disease	5.77	5.61	0.007
End-stage renal disease	2.56	2.49	0.004
Any malignancy	12.47	12.27	0.006
Migraine	4.86	4.89	0.001
Asthma	21.93	22.30	0.009
Chronic obstructive pulmonary disease	13.55	13.85	0.009
Connective tissue disease	6.50	6.46	0.002
Atrial fibrillation	4.97	4.67	0.014
Heart failure	8.74	8.91	0.006
Osteoporosis	25.08	25.52	0.010
Cerebrovascular disease			
Ischemic stroke	11.30	11.40	0.003
Hemorrhagic stroke	0.91	0.91	<0.001
Transient ischemic attack	3.42	3.34	0.004
Acute myocardial infarction	2.65	2.78	0.008
Other ischemic heart disease	25.20	25.77	0.013
Other heart disease	18.18	18.68	0.013
Peripheral artery disease	1.44	1.44	<0.001
Microvascular complications of diabetes			
Neuropathy	10.88	10.58	0.010
Nephropathy	5.79	5.75	0.002
Retinopathy	10.05	10.45	0.013
Alcohol use ^†^	3.48	3.15	0.018
Tobacco use ^†^	0.05	0.07	0.005
Obesity ^†^	0.08	0.08	<0.001
Hypoglycemia	2.73	2.32	0.026
Medication use			
Anti-diabetic medicine			
Metformin	93.98	93.95	0.001
Thiazolidinedione	5.27	5.08	0.008
Alpha-glucosidase inhibitor	15.21	15.39	0.005
Meglitinide	8.33	8.10	0.008
SGLT2i	0.54	0.87	0.039
Insulin	37.45	37.01	0.009
Anti-hypertensive agent			
Calcium channel blocker	69.95	70.21	0.005
ACEI	32.56	32.94	0.008
ARB	72.64	72.91	0.006
Beta blocker	47.96	48.34	0.008
Alpha blocker	13.33	13.04	0.009
Diuretics	67.00	66.71	0.006
Aspirin	73.38	73.90	0.012
P2Y12 inhibitor	32.79	32.93	0.003
Warfarin	5.95	5.55	0.017
Other antiplatelet	25.73	25.45	0.006
NOAC	3.30	3.63	0.018
Lipid-lowering agent			
Statin	72.22	73.20	0.022
Fibrate	15.28	15.04	0.007
Ezetimibe	7.79	8.00	0.008

Data are presented as frequencies or means (SD). ^†^ Confirmed by diagnosis code (International Classification of Diseases, 10th revision). Less than 0.1 (10%) in absolute value of standardized mean difference (SMD) between groups was considered negligible. The mean (SD) standardized difference of all covariates was 1.04% (1.03%). ACEI, angiotensin-converting-enzyme inhibitor; ARB, angiotensin II receptor antagonists; DPP-4i, dipeptidyl peptidase-IV inhibitor; NOAC, novel oral anticoagulant; SD, standard deviation; SGLT2i, sodium-glucose co-transporter 2 inhibitor; SMD, standardized mean difference; SU, sulfonylurea.

**Table 2:** The risk of dementia in DPP-4i use compared with SU use.

	*N*	Events	HR	Lower CI	Upper CI	*p*-Value
Event defined with diagnosis codes						
All-cause dementia	15,104	565	0.66	0.56	0.78	<0.001
Alzheimer’s disease	15,104	367	0.64	0.52	0.79	<0.001
Vascular dementia	15,104	54	0.66	0.38	1.14	0.14
Event defined with diagnosis codes and medication						
All-cause dementia	15,104	184	0.54	0.40	0.73	<0.001
Alzheimer’s disease	15,104	164	0.54	0.39	0.75	<0.001
Vascular dementia	15,104	14	0.46	0.14	1.46	0.18

CI, 95% confidence interval; DPP-4i, dipeptidyl-peptidase IV inhibitor; HR, hazard ratio; *N*, number of patients; SU, sulfonylurea.

**Table 3:** Subgroup analyses according to sex, age, and presence of diabetic microvascular or macrovascular complications.

	*N*	Events	HR	Lower CI	Upper CI	*p*-Value
Male	6601	202	0.60	0.45	0.80	<0.001
Female	8503	363	0.69	0.56	0.85	<0.001
Patients aged ≥75 years	7662	376	0.61	0.50	0.76	<0.001
Patients aged <75 years	7442	189	0.77	0.58	1.03	0.08
Patients with DM microvascular complication	3418	144	0.74	0.53	1.03	0.07
Patients without DM microvascular complication	11686	421	0.64	0.52	0.78	<0.001
Patients with DM macrovascular complication	5487	227	0.67	0.51	0.87	0.003
Patients without DM macrovascular complication	9617	338	0.65	0.52	0.81	<0.001

CI, 95% confidence interval; HR, hazard ratio; *N*, number of patients; DM, diabetes mellitus.
