# Correction: Kim, Young-Gun; Jeon, Ja Young; Kim, Hae Jin; Kim, Dae Jung; Lee, Kwan-Woo; Moon, So Young; Han, Seung Jin. Risk of Dementia in Older Patients with Type 2 Diabetes on Dipeptidyl-Peptidase IV Inhibitors versus Sulfonylureas: A Real-World Population-Based Cohort Study. *Journal of Clinical Medicine* 2019, *8*, 28

**DOI:** 10.3390/jcm8030389

**Published:** 2019-03-20

**Authors:** Young-Gun Kim, Ja Young Jeon, Hae Jin Kim, Dae Jung Kim, Kwan-Woo Lee, So Young Moon, Seung Jin Han

**Affiliations:** 1 Department of Medical Sciences, Ajou University Graduate School, Suwon 16499, Korea; ygkim25@gmail.com; 2 Ministry of Health and Welfare, Gyeonggi Provincial Government, Suwon 16444, Korea; 3 Department of Endocrinology and Metabolism, Ajou University School of Medicine, Suwon 16499, Korea; Twinstwins@hanmail.net (J.Y.J.); jinkim@ajou.ac.kr (H.J.K.); djkim@ajou.ac.kr (D.J.K.); LKW65@ajou.ac.kr (K.-W.L.); 4 Department of Neurology, Ajou University School of Medicine, Suwon 16499, Korea; symoon.bv@gmail.com

The authors wish to make the following corrections to this paper [1].

In Figure 1, all “SGLT-2i” should be replaced by “SU”. Correspondingly, in the figure legend, the definition of “SGLT-2i” should be removed.

After correction, Figure 1 should be displayed as below:

The authors would like to apologize for any inconvenience caused to the readers by these changes.

## Figures and Tables

**Figure 1:**
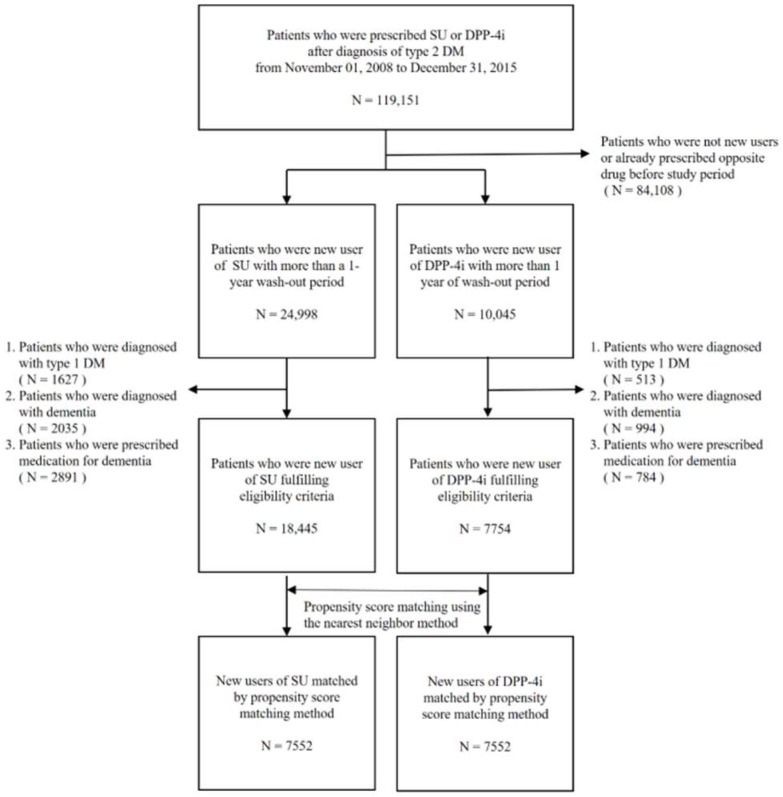
Flow chart of the sample selection process. DM, diabetes mellitus; DPP-4i, dipeptidyl-peptidase IV inhibitor; N, number; SU, sulfonylurea.
